# UV-Assisted Material Extrusion Additive Manufacturing of Double-Base Propellant

**DOI:** 10.3390/polym17060808

**Published:** 2025-03-19

**Authors:** Manman Li, Yuchen Gao, Qionglin Wang, Weitao Yang, Guo-Lin Gao, Zaixing Jiang

**Affiliations:** 1 Xi’an Modern Chemistry Research Institute, Xi’an 710065, China; mml204@163.com (M.L.); yuchengao59@163.com (Y.G.); wangqionglin359@126.com (Q.W.); 2 MIIT Key Laboratory of Critical Materials Technology for New Energy Conversion and Storage, School of Chemistry and Chemical Engineering, Harbin Institute of Technology, Harbin 150001, China

**Keywords:** UV-assisted 3D printing, material extrusion, double-base propellant, UV resin, periodic woodpile structure, performances

## Abstract

Double-base (DB) propellants, renowned for their superior performance and cost-effectiveness, are extensively utilized in both rocketry and artillery applications. During the 3D printing process of double-base propellants, auxiliary solvents play a crucial role in plasticizing the DB propellant mixtures. Consequently, the printed propellants are prone to significant shrinkage and dimensional instability as a result of solvent evaporation post-printing. To address these challenges, we have innovated a UV-assisted material extrusion 3D printing technique that preserves the intended geometries of the DB propellant. The results of our printing trials indicate that incorporating an energetic UV-curable resin as a modifier into the DB propellant paste is highly effective. Ultimately, we successfully fabricated a porous propellant cylinder featuring a periodic woodpile structure. Additionally, the internal structure, mechanical properties, combustion characteristics, and in-barrel ballistic performance of the printed propellants have been thoroughly characterized. Our findings underscore that the UV-assisted material extrusion additive manufacturing process confers exceptional properties to the DB propellant.

## 1. Introduction

The development of gun propellants is essential for enhancing the mobility, range, and muzzle velocity of artillery systems. The traditional manufacturing techniques for propellants encompass specialized processes such as balling, pressing, and rolling. However, existing manufacturing technologies for gun propellants have reached a plateau, limiting the evolution of propellant charges and ammunition [1,2,3]. To overcome this, there is a pressing need for innovative solutions that can expand the design possibilities of gun propellants.

Additive manufacturing (AM), with its ability to produce complex three-dimensional components directly from digital models, offers significant potential for the creation of novel propellant designs that were previously unattainable through conventional methods. In the realm of 3D printing technologies for energetic materials, stereolithography, extrusion-based 3D printing, and three-dimensional printing (3DP) are currently prominent [4]. Direct ink writing (DIW) stands out as a versatile extrusion technique, capable of crafting complex 3D structures using inks or slurries with precisely controlled rheological properties.

Until now, trinitrotoluene/cyclotetramethylene tetranitramine (TNT/HMX) and poly vinylidene fluoride/aluminum (PVDF/Al), along with hydroxyl-terminated polybutadiene-ammonium perchlorate (HTPB-AP) composites, have been successfully employed in DIW for the printing of explosives and rocket propellants [5,6,7,8]. The use of UV-curable energetic composites in 3D printing has also been explored for the fabrication of explosives, solid rocket propellants, and gun propellants [9,10,11]. Michiel et al. [12] demonstrated the 3D printing of gun propellants from an ultraviolet (UV)-curable composite slurry comprising 70% hexogen (RDX), 10% energetic plasticizer, and a UV-curable acrylate binder, achieving a force constant of 1032 J/g. (Force constant is an alternative term for a spring constant, as defined by Hooke’s law. Force constant of a propellant is one of the energy features of a propellant, and also an important ballistic character of the propellant.) Gao et al. [13] further advanced the field by producing composite propellants with higher solid loadings using DIW and UV-curable slurries containing 70% to 80% 2,4,6,8,10,12-hexanitro-2,4,6,8,10,12-hexaazaisowurtzitane (CL-20), resulting in force constants ranging from 1107.43 to 1209.44 J/g. Li et al. [14] introduced acrylate-terminated poly-3-nitratomethyl-3-methyloxetane (APNIMMO), an energetic UV-curable acrylate binder, which increased the force constant of printed gun propellant by 15% at an equivalent solid content. Despite these advancements, propellants with high concentrations of energetic solids still face challenges such as inferior mechanical properties and elevated costs.

Nitrocellulose, a widely utilized binder in propellants, boasts excellent economic viability and mechanical performance. DIW has been used to manufacture nitrocellulose (NC)-based gun propellants through an extrusion process, where NC paste is pre-plasticized with solvents. Zhou et al. [15] assessed the application of DIW with gun propellant paste containing varying amounts of solvent. Wang [16] and Afriat [17] have also explored the printing of gun propellants from diethylene glycol dinitrate/nitroglycerine (DEGDN/NG) paste using screw-driven DIW and vibration-assisted direct-write additive manufacturing, respectively. In these processes, the proportion of added auxiliary solvents typically ranges from 0.3 to 0.6, with 0.4 being the optimal value. However, the extrusion pressure usually reaches several megapascals, which imposes higher requirements on the printer. While increasing the solvent content can improve the flowability of the material, an excessive amount of solvent may cause significant shrinkage in the printed samples.

Leveraging the benefits of UV-curable resins for rapid curing, we have designed and fabricated a double-base propellant containing a UV-curable acrylate resin using more solvent and under lower extrusion pressure, which is printable and can be cured under UV light. Furthermore, we have printed a porous propellant with a periodic woodpile lattice structure using the DIW printer. The performance of this propellant, including its 3D printing method, internal structure, mechanical properties, combustion behavior, and interior ballistic performance, has been thoroughly evaluated.

## 2. Materials and Methods

### 2.1. Material Preparation

The double-base (DB) propellant carpet, comprising 60% nitrocellulose and 40% nitroglycerine/1,5-diazido-3-nitrazapentane (NG/DIANP) serving as the plasticizer, was meticulously crafted and thoroughly dried in our in-house laboratory. An energetic UV-curable oligomer, APNIMMO, was used as UV-curable resin, and the detailed account of the preparation procedure of APNIMMO has been provided elsewhere [14]. Trimethylolpropane trimethacrylate (TMPTA) was used as a reactive diluent and diphenyl (2,4,6-trimethylbenzoyl) phosphine oxide (TPO) as a photoinitiator; both were purchased from Aladdin, Shanghai, China.

### 2.2. Preparation of UV-Curable Paste

For conventional UV-curable systems, the photoinitiator concentration typically ranges from 1 wt.% to 3 wt.%. In this study, we opted for the upper limit of 3 wt.%. This concentration was specifically selected to optimize the curing speed of the extruded filaments during the printing process, ensuring rapid solidification of UV resin in the composite.

The formulation of the UV-curable resin began with the blending of 60 g of APNIMMO, 37 g of TMPTA, and 3 g of TPO in an electric mixer for 1 h. Subsequently, 400 g of the DB propellant carpet, the aforementioned 100 g of UV-curable resin, and 500 g of acetone were combined in a 2.5 L kneader for 3 h. It is crucial to shield the mixture from light throughout the process due to its sensitivity to UV radiation. Upon completion of these steps, the DB-APNIMMO paste is prepared.

For comparative analysis, a DB paste was also produced by kneading 500 g of DB propellant carpet with 500 g of acetone in the same 2.5 L kneader for 3 h.

### 2.3. UV-Assisted DIW Printing Process

**Figure 1:**
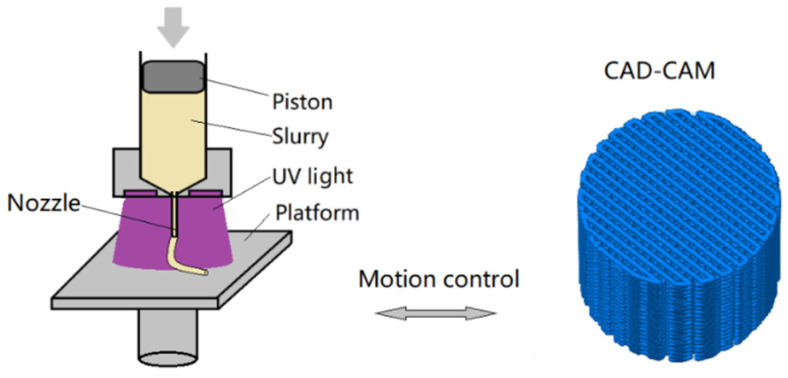
Schematic diagram of DIW 3D printing and print path.

The key advancement in our printing process is the integration of a higher proportion of auxiliary solvent, which substantially lowers the viscosity of the propellant, facilitating extrusion at reduced pressure. Nonetheless, the augmented solvent content resulted in increased shrinkage during printing. To address this, we incorporated a UV-curable binder to assist the printing process. The schematic in Figure 1 delineates the fabrication principle of the UV-assisted DIW technique. The propellant paste, prepared as described, is loaded into a 50 mL precision syringe fitted with a 0.27 mm plastic nozzle. The paste is extruded through this nozzle at a calibrated pressure of 0.5 MPa, ensuring consistent deposition onto an aluminum alloy substrate. Surrounding the nozzle, a 405 nm UV light source with an intensity of 50 mW/cm^2^ is positioned to initiate the photopolymerization of the UV-curable resin within the DB paste. The motion of the XYZ printing platform is orchestrated by G-code, which provides precise control over the deposition path and layering sequence.

The construction of cylindrical structures with a complex woodpile lattice architecture is printed using both the standard DB paste and the DB-APNIMMO paste. The woodpile lattice is built by stacking layers of propellant rods in a specific sequence, with each layer rotated 90 degrees relative to the layer below it, creating an alternating orthogonal pattern that resembles a stack of logs arranged in a crisscross fashion. The printing speed is 10 mm/s, and the fill spacing is adjusted to 0.6 mm.

Upon completion of the DIW process, the DB-APNIMMO cylinders are subjected to a post-print curing regime under the UV light for an extended period of 30 min, enhancing the cross-linking density of APNIMMO. In the final curing stage, the printed propellant cylinders are placed in an oven at 50 °C for a duration of 8 h. This post-processing step is essential for the complete removal of the acetone, resulting in a robust and fully cured propellant lattice.

### 2.4. Characterization

The morphology was observed using a QUANTA FEG 250 (FEI company, Hillsboro, OR, USA) scanning electron microscope (SEM) and a micro-computed tomography (u-CT) system, YXLON FF20 CT (Yxlon International GmbH, Hamburg, Germany).

Tensile tests and compression performances were characterized using a universal mechanical testing machine INSTRON 5969 (Instron Engineering Corporation, Norwood, MA, USA) at a temperature of 20 °C and a loading speed of 10 mm/min.

**Figure 2:**
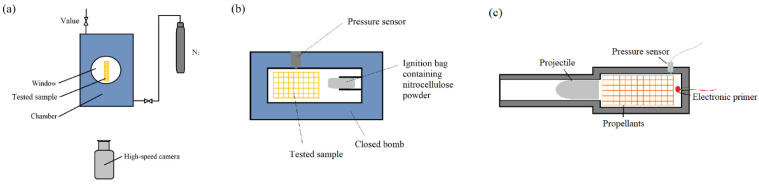
Diagram of combustion test instrument. (**a**) Transparent combustion chamber, (**b**) closed bomb, (**c**) 5.8 mm pistol simulation device.

The combustion performances and internal ballistic performance are detected by transparent chamber, closed bomb, and gun firing, as depicted in Figure 2.

Combustion visualization experiments were conducted in a transparent combustion chamber. The transparent combustion chamber (shown in Figure 2a) was pressurized to 3 MPa and 5 MPa. Ignition of the strand was achieved using a NiCr wire. The burning process of the printed strand of 10 × 5 × 100 mm was captured by a high-speed camera through a viewing window.

The combustion behavior of printed propellant at extremely high pressure was analyzed by a 100 mL closed bomb (shown in Figure 2b). In this process, 12 g of printed propellant cylinders with the woodpile structure shown in Figure 1 were loaded into the bomb and ignited using 1.1 g of nitrocellulose powder with 12.4% nitrogen content. The apparent burn rate of the tested sample was derived from Vieille’s law [18].

The internal ballistic performances were carried out by using a 5.8 mm pistol simulation device (as shown in Figure 2c). For this test, 0.71 g and 1.02 g of 3D printed propellants were loaded in the barrel and ignited by an electric primer. The pressure in the barrel was recorded by a pressure sensor, and the firing process was recorded by a high-speed camera at 4000 frames per second.

## 3. Results and Discussion

### 3.1. Printability and Material Characteristics

**Figure 3:**
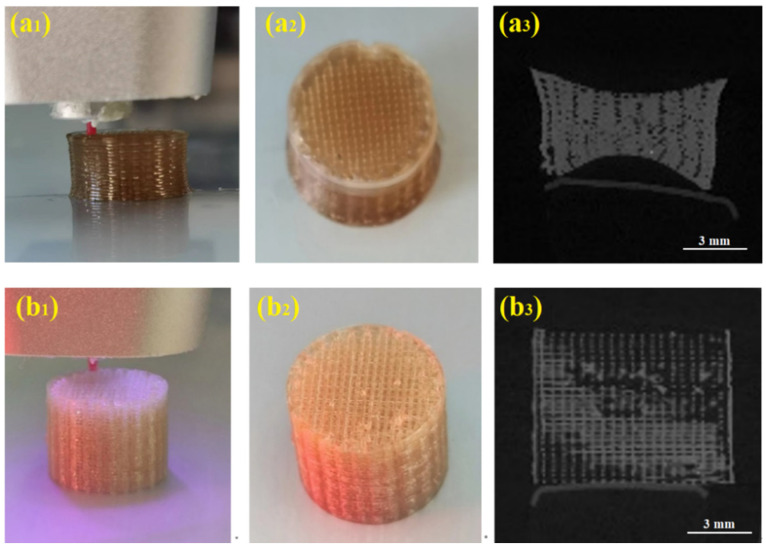
DIW printer depositing propellant pastes into a *d*10× 10 mm cylinder shape. (**a_1_**–**a_3_**) Printing of DB propellant, (**b_1_**–**b_3_**) printing of DB-APNIMMO propellant.

To assess the impact of UV resin on the 3D printing process, cylinders featuring a woodpile structure with dimensions of 10 × 10 mm in diameter and height were fabricated using the DIW technique. Figure 3a_1_–a_3_ illustrate the sequence of the printing: depositing the extruded filament of the DB paste from the nozzle, a photograph of the dried DB cylinder, and a CT scan map. As observed in Figure 3a_1_, the plasticized DB paste was effectively extruded and layered at a pressure of 0.5 MPa and a printing speed of 10 mm/min. However, the cylinder exhibited a slight shrinkage during the printing process, which intensified after the evaporation of acetone, as indicated in Figure 3a_2_,a_3_. An effort was made to mitigate this issue by reducing the acetone content in the DB paste, but the paste became excessively viscous, preventing extrusion through a 0.27 mm nozzle, even with the pressure increased to approximately 1.5 MPa. In conclusion, overcoming the inherent challenges of shrinkage and deformation when printing with plasticized DB paste remains a formidable task.

The challenge of shrinkage and deformation was effectively addressed through the utilization of DB-APNIMMO pastes, which were printed with the support of a rapidly curing APNIMMO resin under UV light irradiation. Figure 3b_1_–b_3_ sequentially present the process: a nozzle dispensing DB-APNIMMO paste under UV light for initial curing, a photograph of the fully cured DB-APNIMMO cylinder, and a CT scan map. It is evident that the DB-APNIMMO pastes, when complemented by the UV-curable APNIMMO resin, achieve a significantly enhanced print quality. The photocuring mechanism of the APNIMMO resin within the DB paste facilitates the preservation of the printed shape, both during and post-printing, even after solvent evaporation. These findings underscore the precision and stability of the 3D features produced by UV-assisted printing with DB-APNIMMO propellants, showcasing a marked improvement in the printing process.

**Figure 4:**
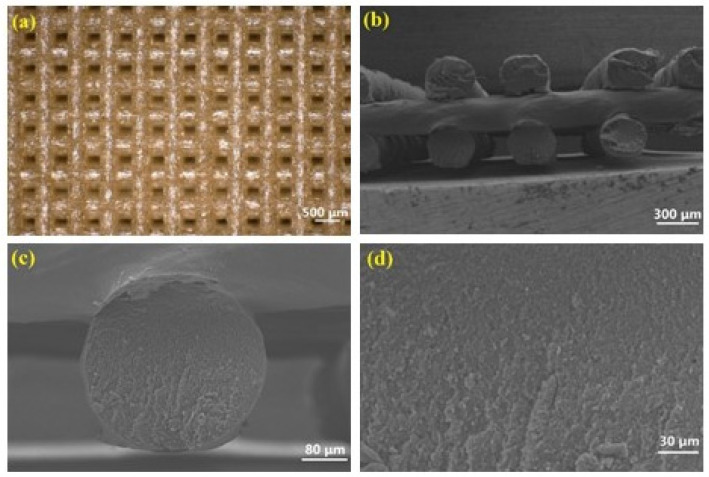
Structure of 3D scaffold: micrograph of scaffold (**a**), SEM image of woodpile lattice (**b**), SEM of the cross section of filament (**c**), SEM of inner structure of filament (**d**).

The microstructural characteristics of the DB-APNIMMO printed architecture, as elucidated by optical microscopy and scanning electron microscopy (SEM), are presented in Figure 4. In Figure 4a, the periodic square cross-sectional morphology of the constructs is evident, with filaments exhibiting a uniform diameter of approximately 0.35 mm and inter-filament spacing of about 0.33 mm. The high-resolution images in Figure 4b further delineate the stacking of the filaments, indicative of a stable assembly process.

Figure 4c provides an enhanced perspective on the filaments, underscoring their ability to maintain a consistent circular cross-sectional profile post-drying, a critical attribute for structural integrity. The cross-sectional SEM micrographs in Figure 4d reveal the absence of microstructural defects such as micro-cracks and micro-voids, which are detrimental to material performance. These findings collectively attest to the superior uniformity and structural integrity of the filaments and the stacked constructs, which are essential for advanced 3D printing applications. The SEM and optical microscopy analyses confirm that the DB-APNIMMO propellants, fabricated via a 3D printing process with UV-curing, exhibit the requisite uniformity and defect-free microstructure. These attributes underscore the suitability of the DB-APNIMMO paste for high-precision printing.

Table 1 presents the key energetic parameters of the propellants, including the force constant (*f*), heat of explosion (*Q*_v_), adiabatic explosion temperature (*T*_v_), and the average molecular weight of the combustion gases. As depicted in Table 1, the incorporation of 20 parts by weight of APNIMMO into the DB propellant formulation results in a reduction of the force constant by approximately 14.2% and a decrease in the adiabatic flame temperature by 23.7%. Since the oxygen content in APNIMMO is lower than that in the DB, this leads to a higher concentration of CO and H_2_ in the gas. Consequently, the average molecular weight of the gas from the DB powder containing APNIMMO is reduced. Our attempts to reduce the APNIMMO content below 20 parts in the DB paste yielded unsatisfactory printing outcomes, underscoring the importance of this threshold in achieving optimal print quality. With a force constant and flame temperature of 973 J/g and 2334 K, respectively, the DB propellant containing 20% APNIMMO aligns with the performance parameters of single-base propellants commonly utilized in firearms and artillery. We have experimented with reducing the content of UV-curable resin, but the printing results were not significantly improved compared to pure DB printing. However, further increasing the content of UV-curable resin would lead to a further decrease in the propellant’s force constant. This reduction in energy would adversely affect the energy density within the limited chamber volume.

Given the balance between printing efficacy and energetic properties, a 20% APNIMMO content in the DB paste is deemed to be a viable and acceptable formulation, offering a promising compromise between the desired energy release and the practical requirements of 3D printing technology in the context of propellant manufacturing.

### 3.2. Mechanical Strength

**Figure 5:**
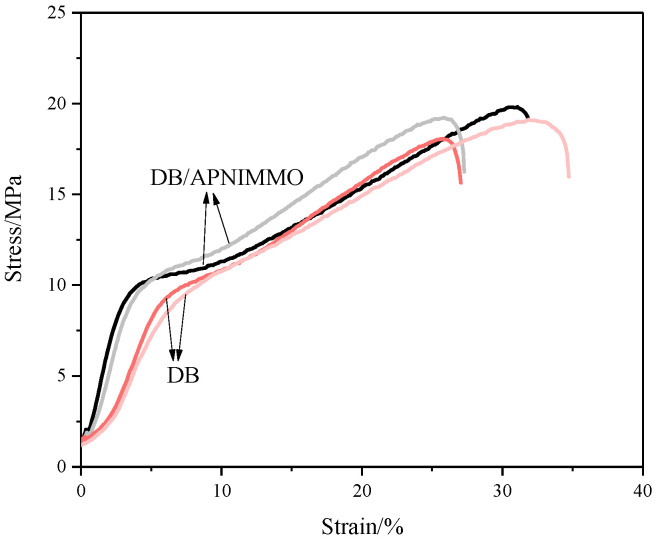
Tensile strength of extruded filaments with/without UV resin from two parallel tests.

DB propellants are renowned for their exceptional mechanical strength [19]. Figure 5 illustrates the stress–strain curves for DB filaments, both with and without the incorporation of APNIMMO resin. Meanwhile, Table 2 enumerates the mechanical properties, including the initial modulus, yield strength, yield strain, ultimate tensile strength, and elongation at break. The graphical profiles in Figure 5 delineate the characteristic behavior of elastic fracture, encompassing stages of elastic deformation, plastic yielding, strain hardening, and eventual fracture.

The tensile strength and elongation rate of the DB material are found to be in alignment with the benchmark data reported by Steinberger [20]. Interestingly, the introduction of APNIMMO resin does not exert a significant impact on these mechanical attributes. The Young’s modulus, an intrinsic material property indicative of stiffness, is notably enhanced in the DB-APNIMMO filament. This increase in the initial modulus suggests a heightened stiffness due to the cross-linking effect of the APNIMMO resin. Conversely, the reduction in yield strain indicates a propensity for the DB propellant to exhibit brittle behavior after the addition of APNIMMO. Further, the deviation in the tensile curves of the same material is the result of multiple factors working together, including the material’s microstructure, sample preparation, and more, which leads to differences in the tensile curves of the same material. As shown in Figure 5, it can be observed that the mechanical curves of the two parallel tests are not entirely consistent. The deviation of breaking strength is 0.3 MPa and 0.5 MPa for DB/APNIMMO and DB, respectively. And, the deviation of elongation at break is 2.4% and 3.7%, respectively.

**Figure 6:**
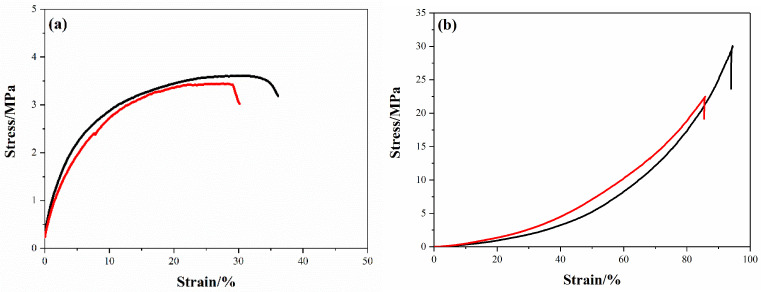
Mechanical strength of stacked filament fabricated by DIW 3D printing from two parallel tests: (**a**) tensile strength, (**b**) compression strength.

Subsequently, the mechanical behavior of the cylinder was characterized. The stress–strain curves derived from the two parallel experiments are presented in Figure 6. Initially, these curves exhibit a linear elastic phase for strains below 5%, succeeded by a plateau indicative of the yielding phase in the porous structure. In two parallel tests, the porous constructs reached failure at strains ranging from 30% to 40%, closely mirroring the behavior of individual filaments. This similarity underscores that the filaments’ toughness and stiffness are pivotal in dictating the tensile properties of the woodpile lattice structure.

Figure 6b presents the compression behavior of two identical cylindrical specimens. As reported earlier, traditional compression curves for DB propellants, fabricated via the screw press technique, have featured a plastic deformation phase (strain below 5%), a yielding phase (5% < strain < 30–40%), and ultimate failure at approximately 40% strain with a strength range of 20–25 MPa [21]. In contrast to these conventional profiles, the stress–strain curves for the tested samples in this study demonstrate a continuous increase with stress, devoid of a distinct yielding phase. It is hypothesized that the rapid strain increases post-lattice densification (at a stress threshold of approximately 30–40%), which accounts for this divergence.

### 3.3. Combustion Performance

**Figure 7:**
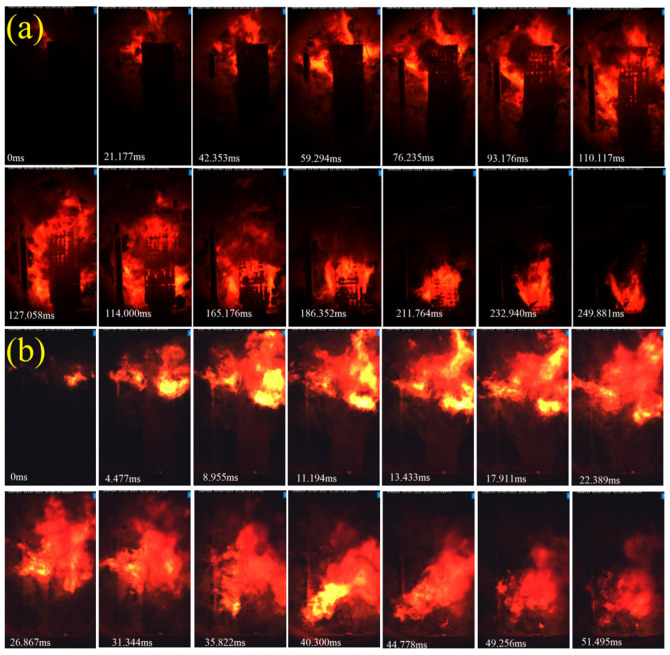
Burning snapshots of architecture at different pressures: 3 MPa (**a**), 5 MPa (**b**).

Porous propellants offer a viable alternative for achieving high burn rates and can serve as monolithic charges, thereby reducing the reliance on traditional granular propellant configurations. A periodic porous strand, with dimensions of 5 mm × 4 mm × 50 mm, was subjected to combustion testing within a transparent-windowed chamber. The combustion process was captured by a high-speed camera. Figure 7 presents a series of snapshots depicting the strand’s combustion behavior at pressures of 3 MPa and 5 MPa. Figure 7a illustrates the phenomenon of in-depth or volumetric combustion under a pressure of 3 MPa, highlighting the penetration of combustion products through the porous lattice structure. Concurrently, the surface combustion reaction rate of the solid propellant is observed to be relatively slow at low pressures, with an approximate 250 ms interval from ignition to the onset of combustion.

At an elevated pressure of 5 MPa, the combustion rate of the solid propellant is significantly enhanced, leading to a more pronounced in-depth combustion as depicted in Figure 7b. Under higher pressure conditions, the propellant strands exhibit a more vigorous combustion process, accompanied by a more luminous flame. The transition from ignition to complete burn-out is markedly expedited, occurring in approximately 50 ms. The combustion process of reaction propagation in propellants is bifurcated into two key stages: gas permeation through the pores and the subsequent combustion of the solid propellant. Initially, the heat-laden gas ignites the filaments within the pores. Once ignited, the filaments’ combustion is predominantly pressure-dependent, akin to conventional solid propellants.

**Figure 8:**
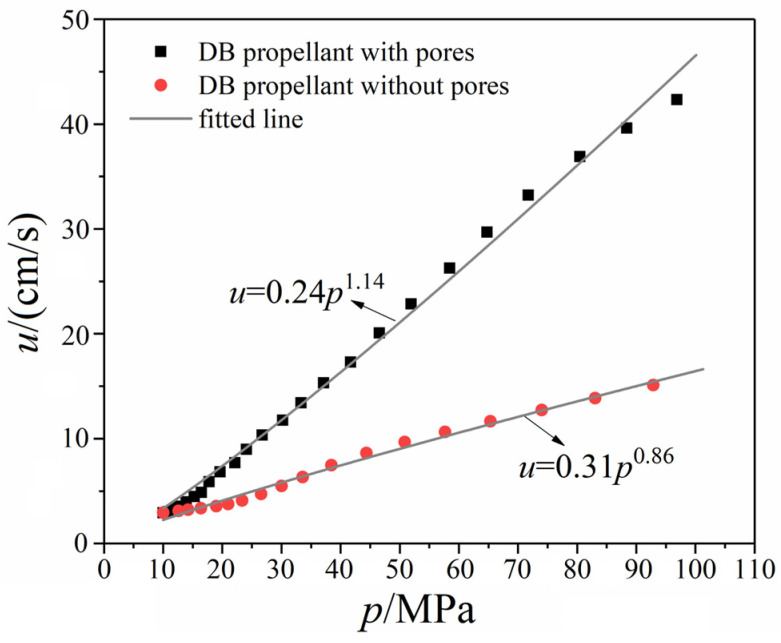
Comparison of apparent burn rate of DB propellants with and without pores.

Figure 8 illustrates the enhanced burn rate of the DB-APNIMMO propellant with integrated porosity. Porous propellants are recognized for their elevated burn rates relative to their non-porous counterparts. Consistent with expectations, the printed porous propellant demonstrates a superior burn rate to the non-porous standard. Specifically, at pressures of 25 MPa, 50 MPa, and 100 MPa, the porous propellant achieved burn rates of 9.7 cm/s, 21.2 cm/s, and 46.3 cm/s, respectively, surpassing the rates of 5.0 cm/s, 9.0 cm/s, and 16 cm/s, respectively, for the non-porous standard at the same pressures. Analysis through Vieille’s law reveals that the porous propellant possesses a higher pressure exponent in its burn rate equation, signifying greater pressure sensitivity. In contrast, the dense DB propellant exhibits a lower pressure exponent.

### 3.4. Interior Ballistic Performance Demonstration

**Figure 9:**
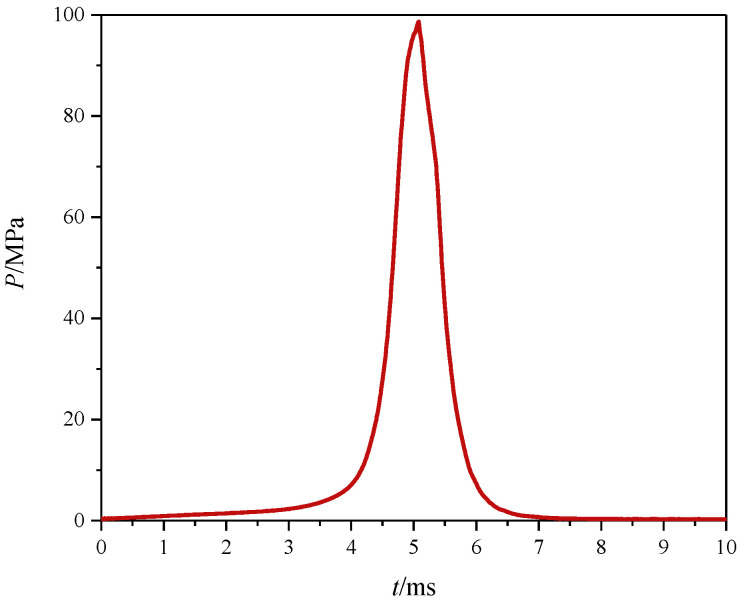
Interior ballistic curves of gun firing.

**Figure 10:**
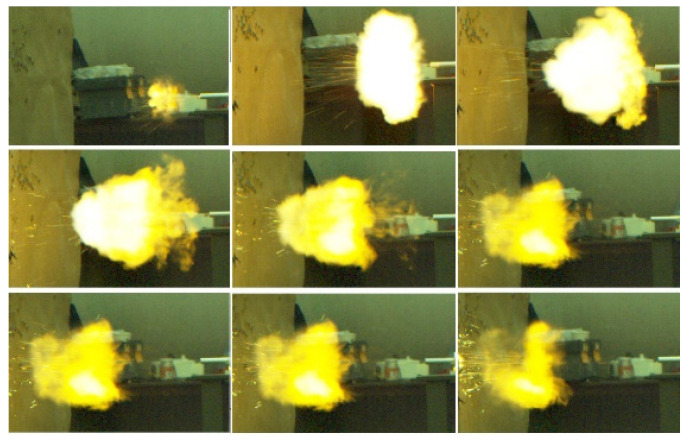
High-speed photographs of gun firing when loading 1.02 g of 3D printed propellant ignited by the electric primer.

In the experimental setup, 1.02 g of porous propellant was loaded into the chamber of the pistol. An electric primer was employed to initiate firing, and the subsequent internal pressure history and the muzzle velocity of the projectile were recorded for subsequent evaluation. Figure 9 presents the internal ballistic performance within the gun barrel, while Table 3 details the internal pressures and the muzzle velocities of the projectiles. Figure 10 captures a high-speed photographic sequence of the firing event. As indicated in Table 3, the chamber pressures achieved 98 MPa, corresponding to muzzle velocities of 31,400 cm/s. These data substantiate the viability and efficacy of employing UV-assisted 3D extrusion technology for the fabrication of double-base propellants as an energy source in artillery systems.

## 4. Conclusions

In summary, this study introduces and substantiates the enhancement of double-base propellant 3D printing quality through the incorporation of photocurable resin. The formulation incorporating APNIMMO’s UV-curable resin demonstrated significant improvements in the manufacturing process of DB propellant. Although the propellant’s force value experienced a 14.2% reduction, the force constant remained within the acceptable operational range. More importantly, the addition of APNIMMO’s resin substantially enhanced the dimensional stability of DB propellant during the printing process, effectively addressing the shrinkage issue commonly encountered in propellant printing. This technological advancement enables the precise fabrication of woodpile-structured propellants with superior structural integrity and dimensional accuracy. The integration of the UV-curable resin APNIMMO into the DB propellant formulation does not adversely impact the material’s strength. Instead, it results in an elevated initial modulus, signifying that the cross-linking induced by APNIMMO augments the composite’s stiffness. This finding underscores the filaments’ toughness and stiffness as pivotal determinants of the tensile integrity within the woodpile lattice structure.

The fabricated porous propellant exhibits distinct combustion characteristics, with volumetric burning behavior at lower pressures and a transition to layer burning at elevated pressures. Empirical burn rate measurements demonstrate that the propellant with a woodpile lattice structure achieves an impressive burn rate of 46.3 cm/s at 100 MPa, which corresponds to a 2.9-fold increase over the non-porous counterpart. The ballistic verification through gun firing tests confirms the practicality of utilizing 3D printed propellants, reinforced with UV resin, as an energy source in barrel weapons.

## Figures and Tables

**Table 1:** The thermal–chemical properties of propellants.

Formula	*f*/(kJ/kg)	*Q*_v_/(kJ/kg)	*T*_v_/K	Average Molecular Weight of Combustion Gas/(g/mol)
DB-APNIMMO	973	2861	2334	20.41
DB	1134	3853	3060	22.43

**Table 2:** Comparison of test data of different strain rates.

Filament Type	Initial Modulus/MPa	Yield Strength/MPa	Yield Strain/%	Breaking Strength/MPa	Elongation at Break/%
DB/APNIMMO	310	8.75	3.41	19.8	30.7
	280	9.21	3.75	19.2	25.9
DB	170	9.26	6.02	18.1	25.7
	160	8.40	6.93	19.1	33.1

**Table 3:** Interior ballistic results.

Propellant Mass/g	*p*/MPa	*V*/(cm/s)
1.02	98	31,400

## Data Availability

The original contributions presented in this study are included in the article. Further inquiries can be directed to the corresponding authors.

## References

[1] Böhnlein-Mauß J., Eberhardt A., Fischer T.S. (2002). Foamed Propellants. Propellants Explos. Pyrotech..

[2] McClain M.S., Gunduz I.E., Son S.F. (2019). Additive manufacturing of ammonium perchlorate composite propellant with high solids loadings. Proc. Combust. Inst..

[3] Wang D., Guo C., Wang R., Zheng B., Gao B., Nie F. (2020). Additive manufacturing and combustion performance of CL-20 composites. J. Mater. Sci..

[4] Bird D.T., Ravindra N.M. (2021). A review: Advances and modernization in U.S army gun propellants. JOM.

[5] Xiao L., Wang Q., Li W., Liu Q., Hao G., Gao X., Ke X., Liu J., Jiang W., Qiao Y. (2018). Preparation and performances of nano-HMX and TNT melt-cast explosives based on 3D printing technology. Acta Armamentarii.

[6] Zhakeyev A., Wang P., Zhang L., Shu W., Wang H., Xuan J. (2017). Additive manufacturing: Unlocking the evolution of energy materials. Adv. Sci..

[7] Bencomo J.A., Iacono S.T., McCollum J. (2018). 3D printing multifunctional fluorinated nanocomposites: Tuning electroactivity, rheology and chemical reactivity. J. Mater. Chem. A.

[8] Chandru R.A., Balasubramanian N., Oommen C., Raghunandan B.N. (2018). Additive manufacturing of solid rocket. J. Propul. Power.

[9] Guo H., Xu S., Gao H.H., Geng X.H., An C.W., Xu C.H., Li Q.B., Wang S., Ye B.Y., Wang J.Y. (2019). CL-20 based ultraviolet curing explosive composite with high performance. Propellants Explos. Pyrotech..

[10] Ye B.Y., Song C.K., Huang H., Li Q.B., An C.W., Wang J.Y. (2020). Direct ink writing of 3D-honeycombed CL-20 structures with low critical size. Def. Technol..

[11] Straathof M.H., van Driel C.A., van Lingen J.N.J., Ingenhut B.L.J., Cate A.T.T., Maalderink H.H. (2020). Development of propellant compositions for vat photopolymerization additive manufacturing. Propellants Explos. Pyrotech..

[12] Rijnders B. (2019). 3D Gradient Printing of Energetic Multi-Materials. Master’s Thesis.

[13] Gao Y., Yang W., Hu R., Zhou J., Zhang Y. (2021). Validation of CL-20-based propellant formulations for photopolymerization 3D printing. Propellants Explos. Pyrotech..

[14] Li M., Yang W., Xu M., Hu R., Zheng L. (2021). Study of photocurable energetic resin based propellants fabricated by 3D printing. Mater. Des..

[15] Zhou M., Nan F., He W., Wang M. (2021). Design and preparation of propellant 3D printer based on extrusion deposition technology. Chin. J. Energetic Mater. (Hanneng Cailiao).

[16] Wang M., Zhou Y., Jin G., Yuan J., Lin X., Nan F., He W. (2022). Extrusion 3D printing technology of double base gun propellants. Chin. J. Energetic Mater. (Hanneng Cailiao).

[17] Afriat A. (2021). Combustion Characteristics of Additively Manufactured Gun Propellants. Master’s Thesis.

[18] Xiao Z.G., Ying S.J., He W.D., Xu F.M. (2013). A novel interior ballistic prediction of gun propellants based on experimental pressure-apparent burning rate model in closed vessel. Adv. Mater. Res..

[19] Tucker J. (2021). A Whole Life Assessment of Extruded Double Base Rocket Propellants. Ph.D. Thesis.

[20] Steinberger R. (1959). The Chemistry of Propellants.

[21] Hu S., Ju Y., Meng H., Zhou C.S. (2011). Effects of strain rate on mechanical properties of DBP under uniaxial compression condition. J. Ballist. (Dandao Xuebao).

